# Economic burden and comorbidities of attention-deficit/hyperactivity disorder among pediatric patients hospitalized in the United States

**DOI:** 10.1186/1753-2000-4-31

**Published:** 2010-12-14

**Authors:** Juliana Meyers, Peter Classi, Linda Wietecha, Sean Candrilli

**Affiliations:** 1 RTI Health Solutions, 200 Park Offices Drive, Research Triangle Park, NC 27709 USA; 2 Eli Lilly and Company, Lilly Corporate Center, DC 6161, Indianapolis, IN 46285 USA; 3 Lilly USA, LLC, Lilly Corporate Center, DC 6161, Indianapolis, IN 46285 USA; 4 RTI Health Solutions, 200 Park Offices Drive, Research Triangle Park, NC 27709 USA

## Abstract

**Background:**

This retrospective database analysis used data from the Healthcare Cost and Utilization Project's Nationwide Inpatient Sample (NIS) to examine common primary diagnoses among children and adolescents hospitalized with a secondary diagnosis of attention- deficit/hyperactivity disorder (ADHD) and assessed the burden of ADHD.

**Methods:**

Hospitalized children (aged 6-11 years) and adolescents (aged 12-17 years) with a secondary diagnosis of ADHD were identified. The 10 most common primary diagnoses (using the first 3 digits of the ICD-9-CM code) were reported for each age group. Patients with 1 of these conditions were selected to analyze demographics, length of stay (LOS), and costs. Control patients were selected if they had 1 of the 10 primary diagnoses and no secondary ADHD diagnosis. Patient and hospital characteristics were reported by cohort (i.e., patients with ADHD vs. controls), and LOS and costs were reported by primary diagnosis. Multivariable linear regression analyses were undertaken to adjust LOS and costs based on patient and hospital characteristics.

**Results:**

A total of 126,056 children and 204,176 adolescents were identified as having a secondary diagnosis of ADHD. Among children and adolescents with ADHD, the most common diagnoses tended to be mental health related (i.e., affective psychoses, emotional disturbances, conduct disturbances, depressive disorder, or adjustment reaction). Other common diagnoses included general symptoms, asthma (in children only), and acute appendicitis. Among patients with ADHD, a higher percentage were male, white, and covered by Medicaid. LOS and costs were higher among children with ADHD and a primary diagnosis of affective psychoses (by 0.61 days and $51), adjustment reaction (by 1.71 days and $940), or depressive disorder (by 0.41 days and $124) versus controls. LOS and costs were higher among adolescents with ADHD and a primary diagnosis of affective psychoses (by 1.04 days and $352), depressive disorder (by 0.94 days and $517), conduct disturbances (by 0.86 days and $1,330), emotional disturbances (by 1.45 days and $1,626), adjustment reaction (by 1.25 days and $702), and neurotic disorders (by 1.60 days and $541) versus controls.

**Conclusion:**

Clinicians and health care decision makers should be aware of the potential impact of ADHD on hospitalized children and adolescents.

## Introduction

Attention-deficit/hyperactivity disorder (ADHD) is a neurobiological disorder that affects children, adolescents, and adults. It is characterized by a persistent pattern of inattention and/or hyperactivity-impulsivity that is more frequent and severe than typically observed in patients at a comparable stage of development. ADHD has been associated with a wide range of lifelong complications, including academic underachievement, conflicting interactions with peers and family members, and low self-esteem, all of which have far-reaching and long-term consequences for individuals [1]. Furthermore, ADHD is a fairly common disorder, with previous studies estimating the prevalence of ADHD in the United States to be about 9% in children and 4.4% in adults [2,3].

Patients with ADHD often suffer from comorbid mood and conduct disorders, which may further complicate treatment. Biederman and colleagues estimated that approximately 30% of pediatric patients with ADHD also had major depression, while Kessler and colleagues found that almost 19% of adult patients with ADHD also had major depression [4,5]. Previous studies have found that between 4.5% and 19.4% of adult patients with ADHD had a concomitant diagnosis of bipolar disorder, compared with about 3.9% in the general population [4-6]. It has been suggested that oppositional defiant disorder (ODD) has a high rate of overlap with ADHD, with between 35% and 40% of ADHD patients also demonstrating signs of ODD [7-10]. Furthermore, patients with ADHD have been found to be at an increased risk of developing substance abuse problems as adults [11,12]. In addition, patients with epilepsy and asthma may be at a greater risk of developing ADHD [13,14].

ADHD has been shown to have serious economic implications for children, families, and society. Patients with ADHD often need long-term care, resulting in significant medical expenditures for prescription drugs and psychotherapy. Previous studies have estimated that children with ADHD have annual health care expenditures that are between US $775 and US $1,330 greater than children without ADHD [15-17]. It is estimated that adults with ADHD have annual expenditures that are approximately US $3,000 greater than adults without ADHD [18].

Despite substantial literature on the costs and economic implications of ADHD, there have been few studies that investigate the impact of ADHD on comorbid conditions and limited studies on the economics of ADHD in the inpatient setting. This study sought to identify the most common primary diagnoses among hospitalized children and adolescents with a secondary diagnosis of ADHD. Patients with these most common primary diagnoses and a secondary diagnosis of ADHD were compared with patients with the same set of primary diagnoses who did not have a secondary ADHD diagnosis to assess differences in patient characteristics, length of hospital stay, and associated costs.

## Methods

Data for this analysis were taken from the Healthcare Cost and Utilization Project (HCUP) Nationwide Inpatient Sample (NIS), a nationally representative inpatient database sponsored by the Agency for Healthcare Research and Quality (AHRQ) [19]. This analysis used data from 2000 to 2006, which represented the most recent years of the NIS available at the time of our study. The NIS is the largest all-payer inpatient care database in the United States and contains data from approximately 8 million hospital stays each year. The data set contains clinical and resource use information typically included in a discharge abstract (e.g., demographics, diagnosis and procedure codes, length of stay [LOS], charges). Financial data in the NIS are presented as charges, which can be converted to costs using facility-specific cost-to-charge ratios. In compliance with the Health Insurance Portability and Accountability Act of 1996 (HIPAA), all data in the database were de-identified to protect the privacy of individual patients, physicians, and hospitals. RTI International's institutional review board determined that this study met all criteria for exemption.

Hospital records for all children (aged 6-11 years) and adolescents (aged 12-17 years) with a secondary diagnosis of ADHD (*International Classification of Diseases, 9th Revision, Clinical Modification *[ICD-9-CM] codes 314.00 and 314.01) were extracted. The 10 most frequent primary diagnoses, based on the first 3 digits of the ICD-9-CM code, were reported for each age group (Table 1). Pediatric ADHD patients with 1 of the 10 most frequent primary diagnoses were selected for inclusion in the ADHD cohorts (i.e., children with ADHD and adolescents with ADHD). Control cohorts included all children and adolescents with no secondary diagnosis of ADHD who also had 1 of the 10 most frequent primary diagnoses among pediatric ADHD patients.

**Table 1:** Summary of the 10 Most Common Primary Diagnoses Among ADHD Patientsa

Patients Aged 6-11 Years					Patients Aged 12-17 Years				
	**Patients With a Secondary ADHD Diagnosis (n = 126,056)**		**Patients Without an ADHD Diagnosis (n = 2,592,204)**			**Patients With a Secondary ADHD Diagnosis (n = 204,176)**		**Patients Without an ADHD Diagnosis (n = 5,130,336)**	
			
**Primary Diagnosis**	**N**	**%**	**N**	**%**	**Primary Diagnosis**	**N**	**%**	**N**	**%**

296: Affective psychoses	30,361	24.09	37,692	0.49	296: Affective psychoses	66,543	32.59	333,817	4.32

313: Emotional disturbances	8,297	6.58	6,584	0.09	311: Depressive disorder NEC	10,589	5.19	68,164	0.88

312: Conduct disturbance NEC	6,810	5.40	8,131	0.11	312: Conduct disturb-ances NEC	9,906	4.85	35,254	0.46

780: General symptoms	6,077	4.82	85,024	1.10	313: Disturb-ances of emotions specific to childhood and adoles-cence	7,970	3.90	19,055	0.25

493: Asthma	5,964	4.73	262,153	3.39	309: Adjustment reaction	7,576	3.71	49,583	0.64

309: Adjustment reaction	4,764	3.78	8,076	0.10	540: Acute appendicitis	5,285	2.59	281,400	3.64

540: Acute appendicitis	3,892	3.09	200,290	2.59	780: General symptoms	4,662	2.28	85,565	1.11

311: Depressive disorder NEC	3,436	2.73	6,493	0.08	300: Neurotic disorders	4,257	2.09	27,432	0.36

345: Epilepsy	2,591	2.06	35,367	0.46	969: Poisoning by psycho-tropic agents	3,853	1.89	29,352	0.38

486: Pneumonia, organism NOS	2,245	1.78	135,420	1.75	250: Diabetes mellitus	3,765	1.84	117,822	1.53

ADHD = attention-deficit/hyperactivity disorder; NEC = Not elsewhere classified; NOS = not otherwise specified.

^a ^Patients with a primary ADHD diagnosis were excluded from the analysis.

Study measures for this analysis included patient and hospital characteristics, LOS, and costs. Patient characteristics included patient age, gender, race, primary expected payer (Medicare, Medicaid, private insurance, self-pay, no charge, other, missing), admission source (emergency room, another hospital, another facility, other, missing), admission type (emergency, urgent, elective, newborn, other, missing), discharge disposition (routine, short-term hospital, skilled-nursing facility, intermediate care facility, another facility, home health care, other, died, missing), and year discharged, while hospital characteristics included geographic region (Northeast, Midwest, South, West, missing), location (urban or rural), teaching status, and bed size. LOS and costs were reported by cohort for each primary diagnosis. Costs were converted from charges, using hospital-specific cost-to-charge ratios, and were updated to 2008 US dollars using the medical care component of the Consumer Price Index.

All data management and analyses were carried out using SAS (version 9.1), Stata (version 11), and SUDAAN (version 9). To account for the complex sampling design of the NIS, appropriate survey-based statistical procedures were employed (i.e., applying sampling weights and using survey procedures to obtain correct variance estimates). Descriptive analyses entailed the tabular display of the mean values, medians, ranges, and standard errors (SEs) of continuous variables of interest (age, LOS, costs) and frequency distributions for categorical variables (e.g., race). Students' t-tests and chi-square tests were used to assess the statistical significance of differences across study measures between the study groups.

In addition to descriptive analyses, we conducted multivariable linear regression analyses to estimate the incremental effect of ADHD on LOS and costs. Regressions were estimated for each primary diagnosis within each age group. The use of regression models to analyze cohort differences in LOS and costs allowed us to control for confounding factors that might not otherwise be accounted for (e.g., gender, geographic region).

LOS and cost models were estimated using a generalized linear model (GLM) with a log-link function and a gamma distribution for the error term to resolve the issue of skewed cost and LOS distributions [20,21]. In addition, the GLM method allowed for adjusted, predicted mean LOS and costs of patients in each study group to be directly calculated in the days or dollars scale, thereby avoiding the issue of potentially biased estimates that may result from retransformation of logged coefficients [22].

Each estimated model included a dichotomous indicator variable, equal to 1 if the patient was in the ADHD cohort and equal to 0 if the patient was not in the ADHD cohort, as well as a vector of underlying patient characteristics (i.e., age, gender, race, primary expected payer, geographic region, hospital teaching status, hospital bed size, hospital location, admission source, discharge destination, and year of discharge). Once a regression model was estimated, predicted values were generated for each patient by cohort. Mean adjusted values were reported, with differences in mean predicted values assessed with t-tests.

## Results and Discussion

### Results

A total of 126,056 children with a secondary diagnosis of ADHD and 204,176 adolescents with a secondary diagnosis of ADHD were identified (Table 1). Among both children and adolescents, the most common primary diagnosis was affective psychoses. Other mental health-related primary diagnoses were common to both age groups (emotional disturbances, conduct disturbances, adjustment reaction, depressive disorder). Additionally, appendicitis and general symptoms were diagnoses common to both cohorts. Among children, diagnoses of asthma, epilepsy, and pneumonia were common, and among adolescents, diagnoses of neurotic disorders, poisoning by psychotropic agents, and diabetes mellitus were common.

Compared with the control cohort, a much higher percentage of patients in the ADHD population were hospitalized with a primary diagnosis of affective disorder (24.09% in ADHD children vs. 0.49% in control children; 32.59% in ADHD adolescents vs. 4.32% in control adolescents). This higher rate in the ADHD cohort was found to be true for all mental health-related hospitalizations, including emotional disturbances (6.58% of ADHD children vs. 0.09% of control children; 3.90% of ADHD adolescents vs. 0.25% of control adolescents), conduct disturbances (5.40% of ADHD children vs. 0.11% of control children; 4.85% of ADHD adolescents vs. 0.46% of control adolescents), adjustment reaction (3.78% of ADHD children vs. 0.10% of control children; 3.71% of ADHD adolescents vs. 0.64% of control adolescents), and depressive disorder (2.73% of ADHD children vs. 0.08% of control children; 5.19% of ADHD adolescents vs. 0.88% of control adolescents). A similar percentage of patients were hospitalized with appendicitis in both cohorts; however, a much higher percentage of ADHD children and a slightly higher percentage of ADHD adolescents were hospitalized with a diagnosis of general symptoms compared with controls (4.85% of ADHD children vs. 1.10% of control children; 2.28% of ADHD children vs. 1.11% of control adolescents). Similarly, a slightly higher percentage of ADHD children had diagnoses of asthma or epilepsy compared with controls (asthma: 4.73% of ADHD children vs. 3.39% of control children; epilepsy: 2.06% of ADHD children vs. 0.46% of control children). Approximately the same percentages of children in the ADHD and control populations were hospitalized with a primary diagnosis of pneumonia (1.78% of ADHD children vs. 1.75% of control children). In adolescents, a slightly higher percentage of patients with ADHD were hospitalized with diagnoses of neurotic disorders or poisoning by psychotropic agents compared with controls (neurotic disorders: 2.09% of ADHD adolescents vs. 0.36% of control adolescents; poisoning by psychotropic agents: 1.89% of ADHD adolescents vs. 0.38% of controls). A similar percentage of adolescents in both cohorts were hospitalized with a primary diagnosis of diabetes mellitus (1.84% of ADHD adolescents vs. 1.53% of control adolescents).

A total of 74,438 children with ADHD and 785,229 children without ADHD had 1 of the 10 most frequent primary diagnoses among ADHD children (Table 2). Children with ADHD were, on average, 6 months older than children without ADHD (mean [SE] 8.74 [0.05] among ADHD children vs. 8.28 [0.02] among control children, P < .001). When compared with control children, a significantly (significance was defined as P < 0.05) higher percentage of ADHD children were male (79.10% of ADHD children vs. 57.50% of control children, P < .001), white (46.01% of ADHD children vs. 35.05% of control children, P < .001), and covered by Medicaid (58.28% of ADHD children vs. 40.46% of control children, P < .001). Additionally, a significantly smaller percentage of ADHD children were admitted to the hospital from the emergency room compared with control children (38.16% of ADHD children vs. 59.23% of control children, P < .001). In both cohorts, most discharges were labeled as routine (94.14% of ADHD children vs. 96.48% of control children), and the highest percentage of patients were from the South (41.31% of ADHD children vs. 37.23% of control children). Additionally, in both cohorts, the majority of children were treated in urban locations (93.58% of ADHD children vs. 86.82% of control children) and more than half were treated in teaching hospitals (61.49% of ADHD children vs. 56.44% of control children) and large bed-size hospitals (63.22% of ADHD children vs. 57.17% of control children). Furthermore, in both cohorts, the distribution of patients was fairly even across all years of admission.

**Table 2:** Demographic and Hospital Characteristics, by Age Group and Cohort

	Patients Aged 6-11 Years					Patients Aged 12-17 Years				
	
	Patients With a Secondary ADHD Diagnosis		Patients Without an ADHD Diagnosis			Patients With a Secondary ADHD Diagnosis		Patients Without an ADHD Diagnosis		
	N	%	N	%	*P *Value	N	%	N	%	*P *Value
**Total sample**	74,438		785,229			124,407		1,047,445		

**Age**										
Mean (SE)	8.74	0.05	8.28	0.02	<.001	14.26	0.04	14.72	0.02	<.001

**Gender**										
Male	58,883	79.10	451,525	57.50	<.001	80,972	65.09	459,199	43.84	<.001
Female	15,426	20.72	315,639	40.20	<.001	43,354	34.85	580,543	55.42	<.001
Missing	129	0.17	18,066	2.30	<.001	81	0.06	7,703	0.74	<.001

**Race**										
White	34,246	46.01	275,189	35.05	<.001	62,186	49.99	470,434	44.91	<.001
Black	11,515	15.47	133,941	17.06	.762	12,088	9.72	110,499	10.55	<.001
Hispanic	5,262	7.07	130,379	16.60	<.001	6,124	4.92	120,415	11.50	<.001
Asian or Pacific Islander	211	0.28	11,179	1.42	<.001	333	0.27	10,477	1.00	<.001
Native American	166	0.22	3,205	0.41	.030	190	0.15	3,909	0.37	<.001
Other	2,445	3.29	27,444	3.50	.280	3,264	2.62	29,546	2.82	.137
Missing	20,593	27.66	203,892	25.97	.408	40,223	32.33	302,164	28.85	.014

**Primary expected payer**										
Medicare	68	0.09	1,108	0.14	.005	217	0.17	1,785	0.17	.004
Medicaid	43,379	58.28	317,705	40.46	<.001	52,562	42.25	352,247	33.63	.582
Private Insurance	26,091	35.05	399,329	50.86	<.001	62,702	50.40	591,369	56.46	.003
Self-pay	1,377	1.85	36,973	4.71	<.001	2,718	2.19	49,774	4.75	<.001
No charge	90	0.12	1,787	0.23	.029	150	0.12	2,523	0.24	.001
Other	3,250	4.37	26,700	3.40	.202	5,597	4.50	47,095	4.50	.006
Missing	184	0.25	1,627	0.21	.556	459	0.37	2,652	0.25	.234

**Admission source**										
Emergency room	28,408	38.16	465,108	59.23	<.001	52,763	42.41	570,497	54.47	.006
Another hospital	3,533	4.75	32,481	4.14	.004	8,057	6.48	61,063	5.83	<.001
Another facility	1,658	2.23	7,966	1.01	.002	3,164	2.54	19,516	1.86	<.001
Other	39,427	52.97	268,892	34.24	<.001	58,447	46.98	379,490	36.23	<.001
Missing	1,411	1.90	10,783	1.37	.213	1,975	1.59	16,879	1.61	.935

**Admission type**										
Emergency	32,191	43.25	409,196	52.11	.803	58,861	47.31	547,153	52.24	<.001
Urgent	24,817	33.34	171,006	21.78	<.001	40,116	32.25	266,490	25.44	.001
Elective	15,205	20.43	102,489	13.05	.519	20,149	16.20	129,372	12.35	<.001
Newborn	176	0.24	938	0.12	.212	355	0.29	1,581	0.15	.170
Other	5	0.01	5	0.00	.120	283	0.23	2,342	0.22	.480
Missing	2,044	2.75	101,595	12.94	<.001	4,642	3.73	100,507	9.60	<.001

**Discharge disposition**										
Routine	70,073	94.14	757,615	96.48	.001	112,805	90.67	966,177	92.24	<.001
Short-term hospital	625	0.84	9,712	1.24	.004	1,557	1.25	12,663	1.21	.625
Skilled-nursing facility	--	--	--	--	--	--	--	--	--	--
Intermediate care facility	--	--	--	--	--	--	--	--	--	--
Another facility	2,685	3.61	6,177	0.79	<.001	8,287	6.66	48,417	4.62	<.001
Home health care	303	0.41	9,458	1.20	<.001	531	0.43	9,232	0.88	<.001
Other	500	0.67	1,434	0.18	<.001	817	0.66	8,025	0.77	.001
Died	9	0.01	474	0.06	<.001	10	0.01	333	0.03	<.001
Missing	243	0.33	360	0.05	.017	399	0.32	2,597	0.25	.021

**Geographic region**										
Northeast	14,964	20.10	173,830	22.14	.504	25,529	20.52	233,768	22.32	.232
Midwest	23,426	31.47	163,678	20.84	<.001	45,597	36.65	291,401	27.82	<.001
South	30,752	41.31	292,322	37.23	.099	43,141	34.68	352,187	33.62	.068
West	5,296	7.11	155,398	19.79	<.001	10,139	8.15	170,089	16.24	<.001

**Location**										
Rural	4,755	6.39	103,181	13.14	.001	9,136	7.34	109,287	10.43	.001
Urban	69,659	93.58	681,728	86.82	.001	115,262	92.65	937,891	89.54	.001
Missing	24	0.03	320	0.04	.590	9	0.01	267	0.03	<.001

**Hospital status**										
Non-teaching	28,644	38.48	341,740	43.52	.835	56,389	45.33	497,552	47.50	.350
Teaching	45,770	61.49	443,170	56.44	.832	68,009	54.67	549,626	52.47	.341
Missing	24	0.03	320	0.04	.590	9	0.01	267	0.03	<.001

**Hospital bed size**										
Small	7,762	10.43	115,576	14.72	.017	12,186	9.80	112,938	10.78	.182
Medium	19,594	26.32	220,380	28.07	.307	28,910	23.24	273,046	26.07	.010
Large	47,058	63.22	448,953	57.17	.017	83,302	66.96	661,195	63.12	.024
Missing	24	0.03	320	0.04	.590	9	0.01	267	0.03	<.001

**Year discharged**										
2000	9,041	12.15	109,581	13.96	.016	13,682	11.00	149,628	14.29	<.001
2001	11,574	15.55	107,463	13.69	.909	17,029	13.69	160,922	15.36	.278
2002	9,100	12.22	107,554	13.70	.206	14,294	11.49	137,383	13.12	.041
2003	11,281	15.16	117,210	14.93	.798	22,485	18.07	163,676	15.63	.065
2004	11,770	15.81	109,515	13.95	.059	19,330	15.54	152,801	14.59	.095
2005	11,917	16.01	127,419	16.23	.512	19,700	15.83	149,631	14.29	.103
2006	9,755	13.10	106,487	13.56	.402	17,887	14.38	133,405	12.74	.022

ADHD = attention-deficit/hyperactivity disorder; SE = standard error.

A total of 124,407 adolescents with ADHD and 1,047,445 adolescents without ADHD had 1 of the 10 most frequent primary diagnoses among ADHD adolescents. Adolescents with ADHD were on average 6 months younger than adolescents without ADHD (mean [SE] 14.26 [0.04] years among ADHD adolescents vs. 14.72 [0.02] years among control adolescents, P < .001). Compared with control adolescents, a significantly higher percentage of ADHD adolescents were male (65.09% of ADHD adolescents vs. 43.84% of control adolescents, P < .001) or white (49.99% of ADHD adolescents vs. 44.91% of control adolescents, P < .001). Additionally, a significantly smaller percentage of ADHD children were admitted to the hospital from the emergency room compared with control children (42.41% of ADHD children vs. 54.47% of control children P = .006). Correspondingly, a significantly smaller percentage of ADHD children had their admission type labeled as emergency compared with control children (47.31% of ADHD children vs. 52.24% of control children, P < .001). In both cohorts, most discharges were labeled as routine (90.67% of ADHD children vs. 92.24% of control children), and patients were fairly evenly distributed over the 4 geographical regions. Additionally, in both cohorts, the majority of children were treated in urban locations (92.65% of ADHD children vs. 89.54% of control children), and more than half were treated in teaching hospitals (54.67% of ADHD children vs. 52.47% of control children) and large bed-size hospitals (66.96% of ADHD children vs. 63.12% of control children). Furthermore, in both cohorts, the distribution of patients was fairly even across all years of admission.

Unadjusted LOS was significantly greater (significant defined as P < .05) for children with ADHD with a primary diagnosis of adjustment reaction (by 1.71 days, P = .029) compared to children without ADHD (Table 3). While not statistically significant, unadjusted LOSs tended to be greater for children with ADHD with a primary diagnosis of affective psychoses (by 0.61 days, P = .102), emotional disturbances (by 0.08 days, P = .928), depressive disorder (by 0.41 days, P = .420), and epilepsy (by 0.56 days, P = .643) compared to children without ADHD. Similarly, while not statistically significant, unadjusted costs tended to be greater for children with ADHD with a primary diagnosis of affective psychoses (by US $51, P = .876), adjustment reaction (by US $940, P = .245), and depressive disorder (by US $124, P = .838) compared to children without ADHD.

**Table 3:** Length of Stay and Costs, by Cohort, Primary Diagnosis, and Age Group

	Length of Stay					Costs				
	
	Patients with a Secondary ADHD Diagnosis		Patients without an ADHD Diagnosis		*P *Value	Patients with a Secondary ADHD Diagnosis		Patients without an ADHD Diagnosis		*P *Value
				
Primary Diagnosis	Mean	Std. Error	Mean	Std. Error		Mean	Std. Error	Mean	Std. Error	
**Patients aged 6-11 Years**										

296 - Affective psychoses	9.41	0.42	8.80	0.52	.102	$7,221	$504	$7,170	$578	.876

313 - Emotional disturbances	10.98	0.71	10.90	0.96	.928	$9,057	$919	$9,479	$948	.596

312 - Conduct disturbance NEC	11.32	0.79	11.82	1.12	.543	$9,967	$1,232	$10,946	$1,392	.185

780 - General symptoms	2.17	0.06	2.33	0.06	.014	$4,336	$231	$5,011	$253	.008

493 - Asthma	2.23	0.05	2.33	0.03	.006	$3,729	$183	$4,182	$152	.001

309 - Adjustment reaction	11.26	1.26	9.55	0.86	.029	$8,806	$1,513	$7,866	$917	.245

540 - Acute appendicitis	2.91	0.11	3.17	0.04	.014	$7,417	$248	$8,147	$141	.002

311 - Depressive disorder NEC	7.80	0.59	7.39	0.42	.462	$6,368	$761	$6,244	$489	.838

345 - Epilepsy	3.75	0.73	3.40	0.17	.643	$8,847	$1,475	$9,618	$659	.607

486 - Pneumonia, organism NOS	2.73	0.09	2.99	0.04	.006	$4,273	$216	$5,077	$152	.001

**Patients aged 12-17 Years**										

296 - Affective psychoses	8.42	0.37	7.38	0.23	<.001	$6,212	$322	$5,859	$274	.044

311 - Depressive disorder NEC	6.54	0.44	5.60	0.25	.005	$5,379	$500	$4,862	$372	.120

312 - Conduct disturbances NEC	11.70	1.22	10.84	1.00	.174	$10,874	$2,175	$9,544	$1,361	.154

313 - Emotional disturbances	9.57	0.84	8.12	0.57	.019	$8,259	$1,268	$6,633	$701	.038

309 - Adjustment reaction	6.97	0.59	5.72	0.38	.002	$5,371	$589	$4,669	$375	.055

540 - Acute appendicitis	2.71	0.08	2.76	0.03	.521	$7,954	$217	$8,181	$109	.235

780 - General symptoms	2.30	0.09	2.39	0.05	.202	$4,894	$253	$5,423	$215	.032

300 - Neurotic disorders	6.68	0.66	5.08	0.24	.006	$5,323	$455	$4,782	$285	.135

969 - Poisoning by psychotropic agents	1.62	0.08	1.62	0.03	.925	$3,577	$174	$3,897	$101	.088

250 - Diabetes mellitus	2.56	0.09	2.56	0.03	.961	$4,177	$198	$4,572	$124	.017

ADHD = attention-deficit/hyperactivity disorder; NEC = Not elsewhere classified; NOS = not otherwise specified; SE = standard error.

Unadjusted LOSs were significantly greater for adolescents with ADHD with a primary diagnosis of affective psychoses (by 1.04 days, P < .001), depressive disorder (by 0.94 days, P = .005), emotional disturbances (by 1.44 days, P = .019), adjustment reaction (by 1.25 days, P = .002), and neurotic disorders (by 1.60 days, P = .006). While not statistically significant, unadjusted LOS tended to be greater for adolescents with ADHD with a primary diagnosis of conduct disturbances (by 0.86 days, P = .174) compared to adolescents without ADHD. Unadjusted costs were significantly greater for adolescents with ADHD with a primary diagnosis of affective psychoses (by US $352, P = .044) and emotional disturbances (by US $1,626, P = .038). While not statistically significant, unadjusted costs tended to be greater for adolescents with ADHD with a primary diagnosis of depressive disorder (by US $517, P = .120), conduct disturbances (by US $1,330, P = .154), adjustment reaction (by US $702, P = .055), and neurotic disorders (by US $541, P = .135) compared to adolescents without ADHD.

Adjusted LOSs were significantly greater for children with ADHD with a primary diagnosis of affective psychoses (by 0.75 days, P < .001), adjustment reaction (by 1.96 days, P < .001), and epilepsy (by 0.18 days, P = .021) (Table 4). While not statistically significant, adjusted LOSs tended to be greater for children with ADHD with a primary diagnosis of emotional disturbances (by 0.48 days, P = .330) and depressive disorder (by 0.43 days, P = .056) compared to children without ADHD. While not statistically significant, adjusted costs tended to be greater for children with ADHD with a primary diagnosis of affective psychoses (by $216, P = .397) and adjustment reaction (by $404, P = .514) compared to children without ADHD.

**Table 4:** Adjusted Length of Stay and Costs, by Age and Diagnosisa,b

	Length of Stay			Costs		
	
	Study Cohort	Control Cohort	*P *Value	Study Cohort	Control Cohort	*P *Value
**Patients Aged 6-11 Years**						

296: Affective psychoses	9.49	8.74	<.001	7,547	7,331	.397

313: Emotional disturbances	11.95	11.47	.330	10,113	10,615	.459

312: Conduct disturbance NEC	11.87	12.10	.622	10,329	11,533	.036

780: General symptoms	2.23	2.45	<.001	4,617	5,255	<.001

493: Asthma	2.28	2.41	<.001	3,979	4,393	<.001

309: Adjustment reaction	11.29	9.33	<.001	8,483	8,079	.514

540: Acute appendicitis	3.10	3.28	<.001	7,630	8,322	<.001

311: Depressive disorder NEC	7.70	7.27	.056	6,188	6,353	.534

345: Epilepsy	3.82	3.64	.021	9,889	10,512	.043

486: Pneumonia, organism NOS	2.67	3.10	<.001	4,387	5,442	<.001

**Patients Aged 12-17 Years**						

296: Affective psychoses	8.28	7.59	<.001	$6,313	$6,253	.583

311: Depressive disorder NEC	6.57	5.85	<.001	$5,415	$5,088	.093

312: Conduct disturbances NEC	12.52	11.40	.062	$11,332	$10,346	.133

313: Emotional disturbances	10.65	9.01	<.001	$8,725	$7,785	.064

309: Adjustment reaction	7.13	5.90	<.001	$5,025	$4,812	.404

540: Acute appendicitis	2.83	2.86	.375	$8,135	$8,323	.014

780: General symptoms	2.34	2.50	<.001	$5,016	$5,715	<.001

300: Neurotic disorders	5.75	5.21	<.001	$4,854	$5,021	.383

969: Poison by psychotropic agents	1.77	1.80	.082	$3,726	$4,105	<.001

250: Diabetes mellitus	2.65	2.62	.499	$4,529	$4,899	<.001

ADHD = attention-deficit/hyperactivity disorder; GLM = generalized linear model; NEC = not elsewhere classified; NOS = not otherwise specified.

^a ^Predicted values derived following GLM regressions for length of stay and costs.

^b ^Covariates estimated in the GLM regressions include age, gender, race, primary expected payer, geographic region, hospital teaching status, hospital bed size, urban or rural location, admission source, discharge destination, year of discharge, comorbidities, and an ADHD indicator flag.

Adjusted LOSs were significantly greater for adolescents with ADHD with a primary diagnosis of affective psychoses (by 0.69 days, P < .001), depressive disorder (by 0.72 days, P < .001), emotional disturbances (by 1.64 days, P < .001), adjustment reaction (by 1.23 days, P < .001), and neurotic disorders (by 0.54 days, P < .001). While not statistically significant, adjusted LOSs tended to be greater for adolescents with ADHD with a primary diagnosis of conduct disturbances (by 1.64 days, P = .062) and diabetes mellitus (by 0.03 days, P = .499) compared to adolescents without ADHD. Additionally, while not statistically significant, adjusted costs tended to be greater for adolescents with ADHD with a primary diagnosis of affective psychoses (by $60, P = .583), depressive disorder (by $327, P = .093), conduct disturbances (by $986, P = .133), emotional disturbances (by $940, P = .064), and adjustment reaction (by $213, P = .404) compared to adolescents without ADHD.

## Discussion

This retrospective database analysis examined demographics, hospital characteristics, LOS, and costs among children and adolescents hospitalized in the United States with a secondary diagnosis of ADHD. The most common primary diagnoses among children and adolescents were identified. Patients with a secondary diagnosis of ADHD were compared with patients without ADHD, using the most commonly observed primary diagnoses. We found that a higher percentage of children and adolescents in the ADHD cohort were male compared with the control cohort and that a lower percentage of children and adolescents in the ADHD group were admitted to the hospital from the emergency room compared with the control cohort. Additionally, a higher percentage of children and adolescents with ADHD had Medicaid listed as their primary expected payer compared with patients without ADHD.

We found that children with ADHD with a primary diagnosis of affective psychoses, adjustment reaction, and depressive disorder had longer LOSs and higher costs compared with children without ADHD. Similarly, adolescents with ADHD with a primary diagnosis of affective psychoses, depressive disorder, conduct disturbances, emotional disturbances, adjustment reaction, and neurotic disorders also had longer LOSs and greater costs compared with adolescents without ADHD. These findings could suggest that children and adolescents with ADHD who are hospitalized for mental disorders may be more difficult to treat compared with children and adolescents without ADHD.

Our study has several limitations common to most retrospective database analyses. First, physician charts were not available to confirm ADHD or other conditions; hospitalizations were identified from diagnosis codes, which, if recorded inaccurately, may cause misidentification of events of interest. Additionally, this study examined only US hospitals; thus, results may not be relevant outside the US setting. Also, only inpatient stays were examined, so results of this analysis may not be generalizable to other care settings.

A number of other studies have used methods similar to those employed in our analysis. Trasande and colleagues studied the burden of obesity on pregnant women and found that obesity was associated with an additional 0.55 inpatient days and an additional US $1,805 in costs [23]. In a study looking at LOS and costs among patients with invasive fungal infections versus matched controls, Menzin and colleagues found that patients with fungal infections had significantly longer LOSs and higher costs versus patients without fungal infections (by 11.4 days and by US $29,281) [24].

## Conclusions

In summary, this study examined common primary diagnoses among children and adolescents with ADHD in an inpatient setting. Patients with a secondary diagnosis of ADHD were compared with patients without ADHD, using the most commonly observed primary diagnoses. Both children and adolescents with ADHD and a primary diagnosis of affective psychoses, adjustment reaction, or depressive disorder had longer LOSs and higher costs compared with patients without ADHD. Additionally, adolescents with ADHD with a primary diagnosis of conduct disturbances, emotional disturbances, and neurotic disorders were found to have longer LOSs and higher costs compared with adolescents without ADHD. Clinicians and other health care decision makers should be aware of the impact that ADHD appears to have on inpatient LOS and costs, when pediatric patients with ADHD present with comorbid conditions in a hospital setting.

## Competing interests

This study was funded by Eli Lilly and Company.

## Authors' contributions

This study was conceived by PC and LW. All authors contributed to the study design and coordination. Database analyses were conducted by SC and JM. The study manuscript was drafted by JM and SC with input from PC and LW. All authors have read and approved the final manuscript.
